# Effects of an integrated ambulatory care program on healthcare utilization and costs in older patients with multimorbidity: a propensity score-matched cohort study

**DOI:** 10.1186/s12877-023-04654-y

**Published:** 2024-01-29

**Authors:** Yu-Tai Lo, Mei-Hua Chen, Tsung-Hsueh Lu, Ya-Ping Yang, Chia-Ming Chang, Yi-Ching Yang

**Affiliations:** 1 Department of Geriatrics and Gerontology, National Cheng Kung University Hospital, College of medicine, National Cheng Kung University, Tainan, Taiwan; 2 Department of Public Health, College of Medicine, National Cheng Kung University, Tainan, Taiwan; 3 Department of Nursing, National Tainan Junior College of Nursing, Tainan, Taiwan; 4 Department of Geriatrics and Gerontology, School of Medicine, College of Medicine, National Cheng Kung University, Tainan, Taiwan; 5 Department of Family Medicine,School of Medicine, College of Medicine, National Cheng Kung University, Tainan, Taiwan

**Keywords:** Healthcare utilization, Integrated care, Multimorbidity, Economics, Taiwan

## Abstract

**Background:**

Population aging has increased the prevalence of multimorbidity, jeopardizing the sustainability and efficiency of healthcare systems. This study aimed to evaluate the effects of an integrated ambulatory care program (IACP) on healthcare utilization and costs among older patients with multimorbidity while accounting for the confounding effects of frailty.

**Methods:**

A retrospective cohort study using propensity matching including patients aged 65 or older with two or more chronic conditions attending the outpatient clinic at our hospital between June 1 and December 31, 2019, was conducted. Exposure was defined as receipt of IACP care. Patients not undergoing the IACP comprised the unexposed group and were matched at a ratio of 1:4 to patients undergoing the IACP group according to sex, age, Charlson Comorbidity Index score, multimorbidity frailty index score, and number of outpatient visits within 6 months before the index date. Outcomes were changes in healthcare utilization and related costs between 6 months before and after receiving IACP care. Multivariate regression analyses were used for data analysis and the Generalized Estimation Equation method was used to fit the regression models.

**Results:**

A total of 166 (IACP) and 664 (non-exposed) patients were analyzed. The mean participant baseline ages were 77.15 ± 7.77 (IACP) and 77.28 ± 7.90 years (unexposed). In univariate analyses, the IACP group demonstrated greater reductions than the unexposed group in the frequency of outpatient visits (-3.16 vs. -1.36, *p* < 0.001), number of physicians visited (-0.99 vs. -0.17, *p* < 0.001), diagnostic fees (-1300 New Taiwan Dollar [NTD] vs. -520 NTD, *p* < 0.001), drug prescription fees (-250 NTD vs. -70 NTD, *p* < 0.001), and examination fees (-1620 NTD vs. -700 NTD, *p* = 0.014). Multivariate analyses demonstrated that patients in the IACP group experienced significant reduction in the frequency of outpatient visits (95% CI: -0.357 to -0.181, *p* < 0.001), number of physicians visited (95% CI: -0.334 to -0.199, *p* < 0.001), and overall outpatient costs (95% CI: -0.082 to -0.011, *p* = 0.01). However, emergency department utilization, hospitalization, and costs did not differ significantly.

**Conclusions:**

Expanding IACPs may help patients with multimorbidity reduce their use of outpatient clinics at the 6-month follow-up, reduce care fragmentation, and promote sustainability of the healthcare system.

**Supplementary Information:**

The online version contains supplementary material available at 10.1186/s12877-023-04654-y.

## Background

Progress in public health and demographic changes are causing a shift in the burden of disease towards chronic noncommunicable diseases, which pose considerable challenges for healthcare systems. According to the World Health Organization (WHO), the global population aged > 60 years will increase to two billion by 2050, and the prevalence of chronic noncommunicable diseases may increase thereafter, particularly among older adults [1]. Multimorbidity, defined as the presence of two or more chronic diseases in the same individual, is associated with complex care needs and leads to increased utilization of healthcare services [2]. Knickman et al. reported that 85% of all healthcare resources are utilized by patients with at least one chronic disease and 65% are spent on patients with multimorbidity [3]. Despite the fact that management of multimorbidity exhausts most healthcare resources, health outcomes are suboptimal [4, 5]. There is an increasing concern that the highly specialized modern medical science and evidence-based guidelines tend to focus on single conditions; however, this approach fails to meet the growing needs of patients with multimorbidity and leads to fragmented medical care that is potentially harmful and duplicated services [6, 7].

According to a systematic review by Desmedt et al., integrated care has received increasing attention because it is considered effective in reducing fragmented services, improving the quality and continuity of care, and controlling healthcare expenditures [8]. According to the WHO integrated care encompasses a wide spectrum of delivery, management, and organizational health services related to health promotion, disease prevention, diagnosis, treatment, disease management, rehabilitation, and palliative care [9]. Integration aims to ensure that people receive a continuum of care at different levels and sites within the health system according to their needs throughout their life course. It also facilitates improvement in patient experiences through care coordination [10].

Although there is a widespread belief that integrated care can control or reduce healthcare utilization and related costs, relatively few studies have evaluated the economic impact of integrated care approaches [8, 11–14]. Earlier studies suggested that integrated care is cost-effective [13] and is likely to reduce costs and improve healthcare outcomes [12]. However, a recent systematic review of randomized clinical trials assessing the effectiveness of integrated models for older patients with chronic diseases in 2022 found that interventions implemented in the models are varied, and it was not possible to determine a single care model as effective [11]. The present body of literature is inconclusive regarding the potential economic impact of integrated care. However, most economic outcomes focus on hospital utilization, including (re)admission rates and emergency visits [13]. There is a scarcity of robust evidence on the economic impact of integrated care on the utilization of outpatient services.

Asia is experiencing a rapidly aging population. In addition to Japan, which has the world’s leading life expectancy and percentage of older people, many Asian countries are experiencing faster demographic changes than European and North American countries, and healthcare expenditures have increased substantially [15]. Therefore, Asia faces the double burden of multimorbidity and the risk of financial sustainability of the healthcare system as many countries provide universal health coverage [16]. For example, a study in Beijing, China, showed that the medical costs of patients with multimorbidity were 3.4 to 5.3 times higher than those with only one chronic condition [17]. Nevertheless, little is known about the impact of integrated care, particularly outpatient-based interventions, on healthcare utilization and costs among older patients with multimorbidity in Asian countries. In four recent systematic reviews on the effectiveness of integrated care models that included 252 studies [8, 11, 12, 14] only two were conducted in Asia [18, 19]. Lin et al. identified 23 integrated care programs in 7 Asian countries; however, only one examined outpatient attendance changes, and only four examined cost changes, indicating that more research is needed for the development of integrated care programs [20].

Moreover, to date, no previous study has considered the confounding effects of frailty on healthcare utilization and costs in relation to integrated care among older adults. Frail older adults are susceptible to adverse health outcomes, including falls, delirium, disability, hospitalization, and mortality, thereby increasing their healthcare utilization [21]. Li et al. demonstrated the significant impact of frailty status on outpatient visits and medical expenditures [22]. To improve research validity and minimize bias, it is essential to collect information on frailty status and effectively manage confounding effects on economic outcomes through proper study design and statistical analyses.

To bridge this knowledge gap, we evaluated the impact of an integrated ambulatory care program (IACP) on healthcare utilization and cost-related outcomes among older adults with multimorbidity at a university hospital in Taiwan.

## Methods

### Study design

This retrospective cohort study was conducted at a 1,193-bed university hospital in Taiwan. This study is reported according to the Strengthening the Reporting of Observational Studies in Epidemiology statement [23] (Supplementary Table 1).

### Data source

The dataset for this study was extracted from hospital medical records by hospital information technologists.

### The integrated ambulatory care program

The IACP in the study hospital was developed and has been offered to patients with multimorbidity since July 1, 2019, to reduce unnecessary utilization of healthcare resources and negative health outcomes. The program was partially supported by the Taiwan National Health Insurance (NHI) [24]. Patients voluntarily participated in the program, and written informed consent was obtained before attending the program. The program involves multidisciplinary teamwork, comprehensive functional assessments, medication reviews, and case management. The multidisciplinary team that implemented the program consisted of case managers, physicians trained as integrated care specialists, and pharmacists. During the first service session, the case manager comprehensively assessed physical, cognitive, nutritional, and mood functions. The comprehensive assessment evaluated the activities of daily living, according to the Barthel Index [25]; cognitive function, according to the Short Portable Mental Status Questionnaire (SPMSQ) [26]; frailty, according to the Clinical Frailty Scale (CFS) [27]; mood, according to the five-item Geriatric Depression Scale (GDS-5) [28]; and nutritional status, according to the Mini Nutritional Assessment–Short Form (MNA-SF) [29]. A pharmacist reviewed the medication regimens of patients. Information on each medication regimen was retrieved from the NHI MediCloud system of Taiwan, which allows medical professionals to access prescription records provided by various hospitals and clinics [30]. The case manager and pharmacist then provided feedback to the physician responsible for the integration. The physician then created a care plan according to the medical condition and preferences of the patient, and the recommendations of the multidisciplinary team. After the initial service session, the patients were followed-up on two occasions by case managers to ensure that no major adverse responses required further evaluation or intervention.

### Study setting and participants

The study included patients who visited the internal, geriatric, or family medicine outpatient clinic at our hospital between June 1 and December 31, 2019. The index date was defined as the date of the first outpatient visit.

Inclusion criteria were as follows: (1) age 65 years or older, (2) more than two chronic conditions, (3) use of more than five medications for chronic disease management, and (4) consultation with more than two physicians for chronic disease management. Patients aged 65 years or older were selected because the pharmacists applied the latest version of the Beers Criteria to identify potentially inappropriate medication and these criteria only apply to patients aged 65 years or older [31]. Patients with more than two chronic conditions were included to meet the criteria for multimorbidity [2]. Patients undergoing treatment with more than five medications were included according to the most commonly reported definition of polypharmacy [32] To evaluate the effectiveness of the complete process of the IACP and to compare healthcare utilization and costs for 6 months before and 6 months after the program, we excluded the following: (1) patients undergoing active cancer treatment (chemotherapy, radiation therapy, and target therapy); (2) patients undergoing clinical trials; (3) patients with no outpatient utilization at the study hospital at 6 months after the index date; (4) patients who died within 6 months after the index date; (5) patients extreme medical costs; (6) patients lost to follow-up after the index date (6) received the program during the outcome evaluation period (between January 1 and June 30, 2020).

### Exposed and unexposed group

Exposure was defined as receiving care under the IACP between June 1 and December 31, 2019. Patients in the unexposed group included those who were not in the IACP and were matched using a propensity score matching algorithm at a ratio of 1:4 to those in the IACP group using five variables: sex, age, Charlson Comorbidity Index (CCI) score [33], multimorbidity Frailty Index (mFI) score [34], and number of outpatient visits within 6 months before the index date. The matching was processed using a greedy nearest neighbor algorithm with a caliper of 0.2 times the standard deviation of the logit of the propensity score, with a random matching order and without replacement.

#### Outcomes

The primary outcomes were changes in outpatient service utilization, including the number of outpatient clinic visits, number of physicians visited, and outpatient service costs between 6 months before and 6 months after the commencement of the program. We measured the overall costs of the outpatient services and subsidiaries, including diagnostics, drugs, prescription services, treatment, and examination fees.

Secondary outcomes included changes in the number of emergency department (ED) visits, hospitalizations, length of hospitalization (days), costs of ED visits, and costs of hospitalizations between 6 months before and 6 months after the commencement of the program. We measured only the overall costs of ED visits and hospitalizations.

Taiwan’s NHI system has adopted a single-payer system that primarily uses fee-for-service payments for medical services, examinations, and medications [35]. This study measured reimbursement payments from the NHI for general diagnoses and treatment, medical consultations and operations, and related expenses. We did not include out-of-pocket fees in this study.

#### Baseline characteristics and covariates

Baseline data on demographic characteristics, namely age, sex, and place of residence, were collected upon the inclusion of participants (index date). Furthermore, clinical data, including body mass index (BMI), CCI scores for comorbidity status [33], number of chronic disease diagnoses, and mFI score for frailty status [35]. Potential covariates included age, sex, distance between home and hospital, comorbidities, frailty status, number of chronic diseases, and baseline use of outpatient services. The distance between home and hospital was categorized according to whether the distance required more than 30 min of driving by car (yes or no).

### Statistical analysis

Continuous variables are presented as means with standard deviations, and categorical variables are expressed as counts with percentages. Comparisons of variables between the exposed and unexposed groups were performed using the chi-square test or Fisher’s exact test for categorical variables and the 2-sample t-test or Mann–Whitney U test for continuous variables. Univariate analysis of the outcomes between 6 months before IACP and 6 months after IACP or without IACP was performed using paired t-tests.

To adjust for the potential confounding effect of covariates and assess the independent effect of IACP on outcomes, we used (1) multivariate Poisson regression analyses for the number of outpatient visits, number of physicians visited, and length of hospital stay; (2) multivariate linear regression analyses for the costs of outpatient services, ED visits, and hospitalizations; and (3) negative binominal regression analyses for the number of ED visits and hospitalizations. In addition, to account for the intercorrelations of data collected from exposed and unexposed participants 6 months before and 6 months after the index date, we used a Generalized Estimation Equation (GEE) to fit the Poisson, linear, and negative binomial regression models. Medical costs in New Taiwan dollars (NTD) were expressed after the logarithmic transformation of the original values in the multivariate analyses.

All the tests were two-tailed. Statistical significance was defined as *p* < 0.05. All statistical analyses were performed using SAS software (version 9.4; SAS Inc., Cary, N.C., USA).

### Ethical approval

The study protocol was approved by the Institutional Review Board of the National Cheng Kung University Hospital (A-ER-109-311).

## Results

### Characteristics of the study population

**Fig. 1:**
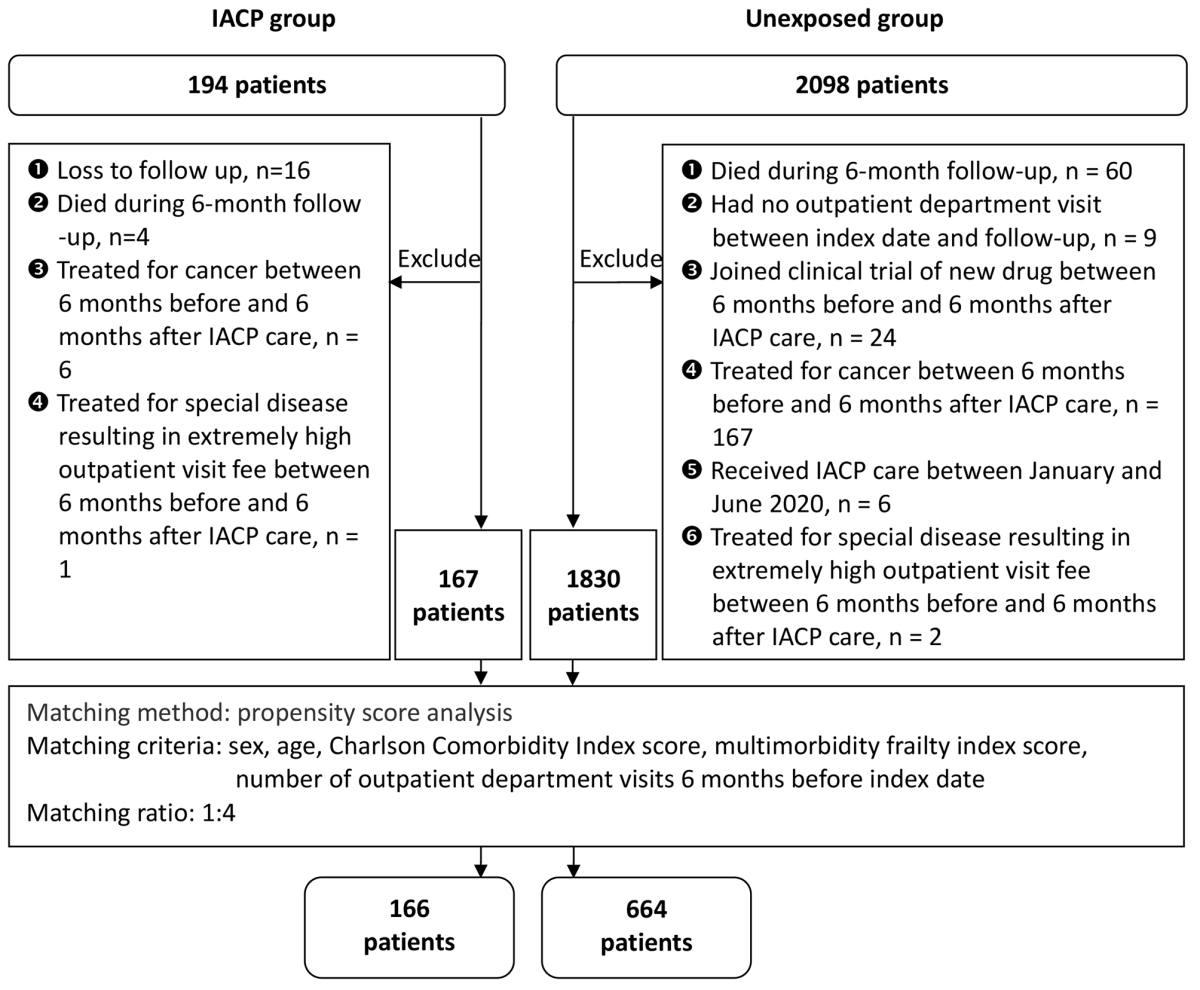
Patient selection flowchart

From June 1 to December 31, 2019, 194 patients participated in the IACP and 2,098 did not participate in the IACP. Following the application of the exclusion criteria, 167 older patients were included in the IACP group and 1,830 in the unexposed group. Each IACP participant was matched 1:4 ratio to the unexposed group. No match was obtained for one patient in the IACP group, who was excluded. A total of 830 patients were enrolled in the final analysis: 166 in the IACP and 664 patients in the unexposed group. The mean age was 77.25 ± 7.87 years, and 48.3% were male. The patients presented a high degree of comorbidity (mean CCI:3.70 SD:2.32) and mild frailty (mean mFI:0.10, SD:0.06). A flowchart of the patient selection process is shown in Fig. 1.

Initially, 16 patients participated in the IACP; however, they were lost to follow-up and were excluded. A comparison of the demographic characteristics and clinical conditions of the IACP participants included in the study and those lost to follow-up is presented in Supplementary Table 2. The analysis revealed no significant differences in demographic characteristics or clinical conditions between the participants included in the study and those lost to follow-up.

A comparison of the demographic characteristics and clinical conditions between the exposed and unexposed groups is presented in Table 1. Compared with the unexposed group, the IACP group had a significantly higher percentage of patients with diabetes (52.4% vs. 38%, *p* = 0.001) and a lower percentage of patients with chronic pulmonary diseases (13.3% vs. 22.7%, *p* = 0.008). No significant differences in the other variables were observed between the IACP and unexposed groups.

**Table 1:** Baseline demographic and clinical characteristics of study participants

Variables	IACP (*n* = 166)	Unexposed (*n* = 664)	*p* value	ASMD^#^
**Demographic characteristics**				
Age (years), mean (SD)	77.15 (7.77)	77.28 (7.90)	0.850	-0.017
Range (min–max)	65–94	65–99		
Sex, male, n (%)	82 (49.4)	319 (48.0)	0.755	-0.027
Home to hospital distance ≤ 30 min car drive, n (%)	151 (91.0)	584 (88.0)	0.277	
**Outpatient department visits**	9.07 (4.21)	9.27 (4.12)	0.566	-0.048
**Clinical characteristics**				
Charlson Comorbidity Index, mean (SD)	3.72 (2.48)	3.70 (2.27)	0.909	0.010
Chronic diseases, mean (SD)	8.86 (3.39)	8.78 (3.18)	0.704	
Multimorbidity frailty index score, mean (SD)	0.10 (0.06)	0.10 (0.06)	0.746	-0.029
BMI, mean (SD)	24.31 (3.86)	24.50 (3.86)	0.586	
Diseases, n (%)				
Diabetes	87 (52.4)	252 (38.0)	0.001*	
Diabetes with end-organ damage	78 (47.0)	316 (47.6)	0.889	
Moderate-to-severe renal disease	76 (45.8)	327 (49.2)	0.425	
Peptic ulcer disease	38 (22.9)	129 (19.4)	0.320	
Cerebral vascular diseases	36 (21.7)	135 (20.3)	0.699	
Any tumor	25 (15.1)	117 (17.6)	0.434	
Dementia	25 (15.1)	100 (15.1)	1.000	
Chronic pulmonary diseases	22 (13.3)	151 (22.7)	0.008*	
Congestive heart failure	22 (13.3)	111 (16.7)	0.278	
Myocardial infarction	4 (2.4)	37 (5.6)	0.103	
Hemiplegia	3 (1.8)	6 (0.9)	0.324	
Peripheral vascular disease	2 (1.2)	23 (3.5)	0.146	
Metastatic solid tumor	2 (1.2)	4 (0.6)	0.422	
Mild liver disease	1 (0.6)	26 (3.9)	0.062	
Connective tissue diseases	1 (0.6)	22 (3.3)	0.091	
Moderate-to-severe liver disease	1 (0.6)	5 (0.8)	0.838	
Lymphoma	0	4 (0.6)	-	
Leukemia	0	3 (0.5)	-	
AIDS	0	2 (0.3)	-	

^#^ASMD, absolute standardized mean difference

### Impact of the integrated ambulatory care program

**Fig. 2:**
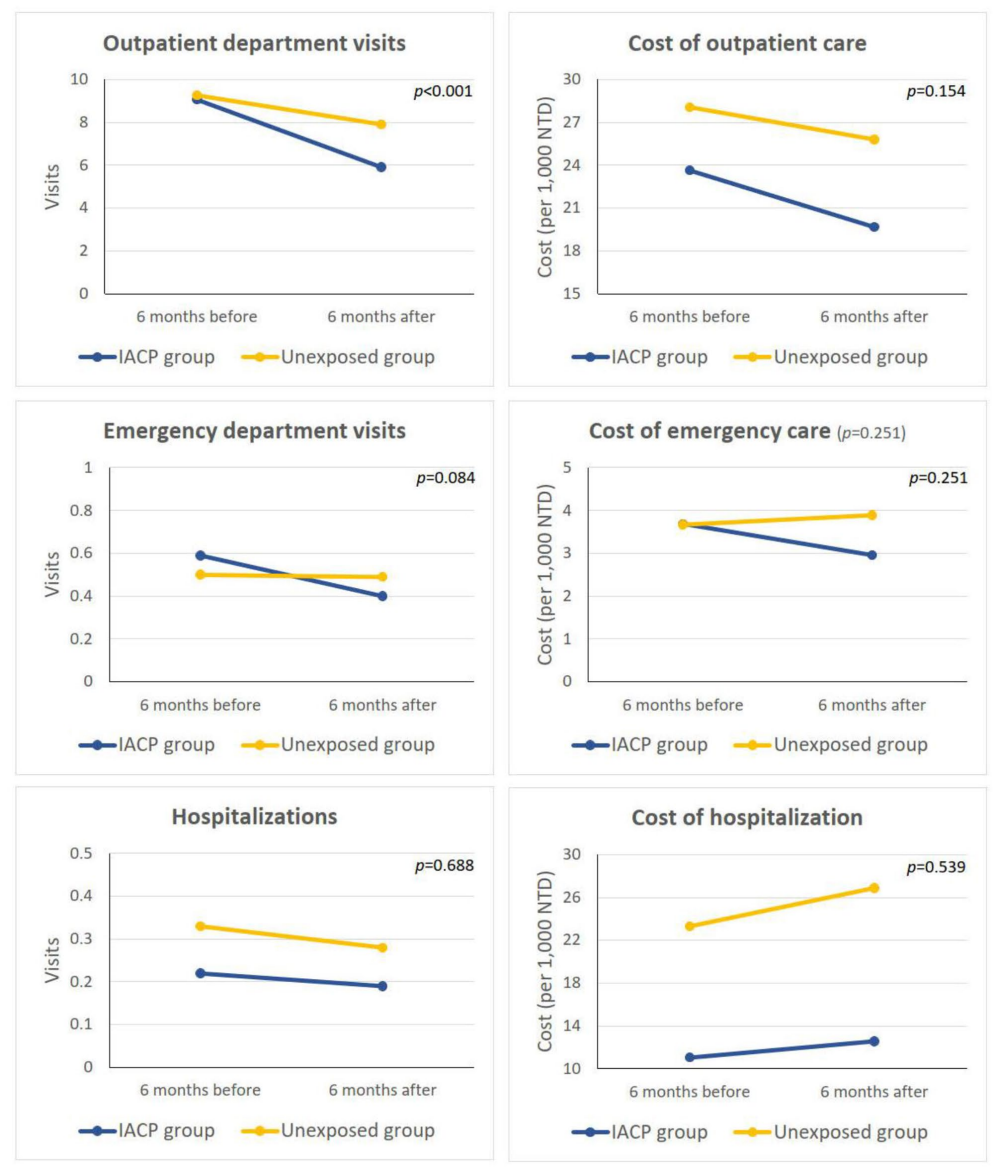
Univariate paired t-test results for outcome variables

The results of the univariate analysis of the outcome differences between 6 months before and 6 months with or without IACP are presented in Table 2; Fig. 2. Compared with the unexposed group, the IACP group experienced significant reductions in the frequency of outpatient visits (-3.16 vs. -1.36, *p* < 0.001) and the number of physicians visited (-0.99 vs. -0.17, *p* < 0.001). Reductions in the frequency of ED visits, hospitalizations, length of hospital stay, overall outpatient and ED care costs were also observed; however, these effects were not significant. Despite no significant reduction in the overall outpatient costs, the IACP group showed significant reductions in diagnostic costs, drug prescriptions, and examination fees. In contrast, hospitalization costs increased 6 months after the index date in both the IACP and unexposed groups; however, the differences were not significant between the two groups. The results of the multivariate analyses using regression models with the GEE method are presented in Table 3. After controlling for several confounding variables, patients in the IACP group experienced significant reduction in frequencies of outpatient visits (β=-0.269, 95% CI: -0.357 to -0.181, *p* < 0.001), number of physicians visited (β=-0.266, 95% CI: -0.334 to -0.199, *p* < 0.001), and overall outpatient costs (β=-0.046, 95% CI: -0.082 to -0.011, *p* = 0.01). Although reductions in the frequency of ED visits, length of hospital stay, and overall ED care costs were observed, these differences were not significant. The frequency and cost of hospitalization tended to increase 6 months after the index date in the IACP group, but no significant effects were found.

**Table 2:** Univariate analysis of outcomes between 6 months before and 6 months after IACP or without IACP

	IACP group (*n* = 166)			Unexposed group (*n* = 664)			*p* value
	6 months before	6 months after	difference	6 months before	6 months after	difference	
Outpatient department visits	9.07 (4.21)	5.91 (4.16)	-3.16 (3.52)	9.27 (4.12)	7.91 (4.25)	-1.36 (3.60)	< 0.001*
Number of physicians visited	3.68 (1.69)	2.69 (1.54)	-0.99 (1.27)	3.70 (1.47)	3.53 (1.56)	-0.17 (1.20)	< 0.001*
Overall Costs of outpatient care^#^	23.65 (13.84)	19.68 (14.98)	-3.97 (11.94)	28.07 (28.61)	25.81 (29.10)	-2.26 (19.86)	0.154
Diagnostic fee	4.78 (1.52)	3.48 (1.52)	-1.30 (1.25)	3.89 (1.39)	3.37 (1.58)	-0.52 (1.24)	< 0.001*
Drug fee	10.27 (7.59)	9.70 (9.21)	-0.57 (6.59)	15.33 (24.31)	13.84 (24.77)	-1.49 (16.17)	0.252
Prescription service fee	0.80 (0.43)	0.54 (0.40)	-0.25 (0.27)	0.86 (0.44)	0.79 (0.50)	-0.07 (0.28)	< 0.001*
Treatment fee	0.87 (1.50)	0.68 (1.94)	-0.20 (1.93)	1.17 (3.08)	1.36 (5.05)	0.19 (4.56)	0.091
Examination fee	5.12 (4.38)	3.52 (3.21)	-1.61 (4.10)	4.77 (4.52)	4.08 (3.99)	-0.70 (4.92)	0.014*
Others	1.80 (7.24)	1.76 (4.63)	-0.04 (5.67)	2.03 (4.87)	2.37 (6.09)	0.34 (6.17)	0.452
Emergency department visits	0.59 (1.16)	0.40 (0.84)	-0.19 (1.21)	0.50 (1.17)	0.49 (1.15)	-0.02 (1.12)	0.084
Overall Cost of emergency care^#^	3.69 (9.17)	2.96 (7.27)	-0.73 (9.37)	3.67 (8.42)	3.89 (9.79)	0.22 (10.12)	0.251
Hospitalizations	0.22 (0.49)	0.19 (0.46)	-0.03 (0.62)	0.33 (0.71)	0.28 (0.66)	-0.05 (0.76)	0.668
Length of hospital stay (days)	1.78 (5.37)	1.71 (6.15)	-0.07 (7.17)	2.48 (6.66)	2.89 (10.22)	0.41 (10.21)	0.481
Overall Cost of hospitalization^#^	11.06 (35.62)	12.58 (66.12)	1.53 (73.08)	23.31 (64.14)	26.89 (105.58)	3.57 (111.51)	0.774

^#^ per 1,000 New Taiwan Dollar

**Table 3:** Generalized estimating equation with regression models on the effect of integrated ambulatory care program on healthcare utilization and related costs

Outcome	B	95% CI	*p*	Model
Outpatient department visits	-0.269	-0.357–-0.181	< 0.001*	Poisson
Number of physicians visited	-0.266	-0.334–-0.199	< 0.001*	Poisson
Overall cost of outpatient care^#^	-0.046	-0.082–-0.011	0.010*	Linear
Diagnostic fee	-0.067	-0.090–-0.045	< 0.001*	Linear
Drug fee	0.018	-0.021–0.056	0.364	Linear
Prescription service fee	-0.132	-0.165–-0.100	< 0.001*	Linear
Treatment fee	-0.308	-0.555–-0.061	0.015*	Linear
Examination fee	0.031	-0.105–0.167	0.655	Linear
Others	-0.082	-0.343–0.178	0.535	Linear
Emergency department visits	-0.386	-0.789–0.017	0.061	Negative binomial
Cost of emergency care^#^	-0.160	-0.530–0.211	0.398	Linear
Hospitalizations	0.068	-0.456–0.591	0.799	Negative binomial
Length of hospital stay	-0.194	-0.878–0.491	0.579	Poisson
Cost of hospitalization^#^	0.084	-0.300–0.468	0.668	Linear

Main effects (reference group): group (unexposed group), period (previous 6 months), sex (female), age, Charlson comorbidities index score, number of chronic diseases, multimorbidity frailty index, distance (≥ 30 min)

Correlations matrix: exchangeable

^#^cost + 1 and take Log_10_ to analysis

## Discussion

This study presents empirical results on the impact of implementing the IACP in Taiwan, including results on healthcare use, particularly outpatient attendance, and costs among older adults with multimorbidity. Our results showed that at the 6-month follow-up, program participants had a significant decrease in the frequency of outpatient visits, the number of doctors visited, and the overall cost of outpatient services. Therefore, the IACP may improve fragmented care and economic sustainability in outpatient settings for older patients with multimorbidity.

Excessive outpatient clinic visits among patients with multimorbidity is a major problem in Taiwan. Although most healthcare services are covered by the NHI, high accessibility, low co-payment, and fee-for-service payment schemes result in high healthcare service utilization and disjointed care [36]. On average, older adults in Taiwan attend outpatient services nearly 30 times per year [37]. Therefore, since 2019, the NHI has encouraged and supported hospitals in establishing integrated ambulatory care services [24]. To the best of our knowledge, this is the first cohort study to explore the economic impact of an IACP in Taiwan.

Our finding that the number of outpatient clinic visits by IACP participants was reduced by 3.16 in 6 months suggests that the program may reduce the unnecessary use of outpatient services. Previous studies have demonstrated a similar effect of integrated care programs. Leung et al. demonstrated that case management in a randomized controlled trial reduced outpatient attendance [38]. In a UK study, Goldzahl et al. evaluated the effect of multi-disciplinary group meetings for the discussion of high-risk older patients on healthcare utilization, and found that an integrated care intervention reduced the probability of visiting primary care nurses [39]. Case management and multi-disciplinary teamwork are important components of the program.

Multivariate analysis revealed that the IACP participants exhibited a significant decrease in the overall cost of outpatient services during the 6-month follow-up period. However, our study did not measure the costs associated with out-of-pocket payments, transportation, forgone earnings, productivity losses resulting from treatment, or time spent during informal caregiving. Considering the reduction in 3.16 outpatient clinic visits observed 6 months after starting the program, it is likely that costs related to transportation, earnings forgone, productivity losses, and time during informal caregiving were also saved after the program.

Wei et al. reported that an integrated geriatric outpatient clinic reduced the annual costs of outpatient care and hospitalization by the time of the 1- and 2-year follow-up. Although our findings are consistent with those reported by Wei et al. on the reduction of outpatient care costs, hospitalization costs were not reduced at the 6-month follow-up in our study. Ye et al. examined the effectiveness of a pilot integrated care model (Louhu) among older Chinese adults and found no impact on hospitalization costs at 1 year [40]. An explanation could be that the average number of hospitalizations among the participants in our study was less than 0.4 in 6 months. Therefore, our study may have been underpowered to determine this outcome. Further research with a larger sample size or participants with a higher risk of admission (e.g., participants recently discharged from the hospital) is required to determine the effect of the program on the prevalence of hospitalization among older patients with multimorbidity.

The three aims of integrated care are to improve health and quality of care and reduce costs [12]. However, despite the increasing attention that integrated care has received, evidence indicates that reported programs are heterogeneous, and there is currently no universally accepted single best practice model or set of guidelines for implementing integrated care. Our study focused on older patients with multimorbidity, a group that experienced the detrimental effects of a fragmented healthcare system. This group has the greatest potential for improvement in terms of quality, cost-effectiveness, and efficiency.

### Limitations and strengths

This study has several limitations, indicating the need for caution when interpreting the findings. First, our study is susceptible to selection bias because we excluded patients who died or were lost to follow-up. Despite the efforts of our case managers to contact participants who refused further follow-up and suggested rescheduling appointments, the patients still preferred to forego further services. Nevertheless, there were no significant differences in the demographic characteristics and clinical conditions between patients included in the study and those lost to follow-up. Second, we did not consider healthcare use at other medical facilities because we could only access the medical records at the study hospital. Third, this study involved patients from a single university hospital, and our findings may not be generalizable to patients receiving health services at other institutions. Fourth, the cohort study design made it impossible to create a comparable unexposed group; therefore, residual confounding and confounding by indication might have affected the interpretation of the results. However, we addressed this problem in both the study design and data analysis stages by adopting propensity score matching and the GEE method with regression models. Propensity score matching was used to eliminate confounding factors due to the measured covariates, particularly the effect of frailty status. Furthermore, we measured various covariates that were not considered in previous studies, such as frailty status, distance from home to the hospital, and baseline healthcare utilization. Our outcomes were repeatedly measured 6 months before and 6 months after the index date in both groups; therefore, the data were highly correlated. Using GEE methods with regression models helps improve the validity and precision of data analyses [41]. Finally, the retrospective data did not cover changes in the participant function or patient-related outcome measures, which are helpful in conducting different economic evaluations, such as cost-utility or cost-benefit studies, and strengthen the value of the study.

Despite these limitations, the strengths of this study include the evaluation of economic outcomes, which are lacking in the current literature. The positive results obtained in this study can serve as a reference for other Asian countries wishing to develop integrated care for older adults with multimorbidity, and are therefore directly relevant to program managers and healthcare professionals. Our results can serve as a reference for future studies aimed at elucidating the influence of outpatient-based integrated care interventions on the economic outcomes of older patients with multimorbidity. From a public health efficiency perspective, integrated care programs may serve as a promising solution to address the fragmentation and growing healthcare expenditures related to population aging.

## Conclusions

Population aging has increased the prevalence of multimorbidity, jeopardizing the sustainability and efficiency of healthcare systems. Hospital-based multidisciplinary IACP reduced outpatient visit frequency, physician visits, and outpatient service costs among older patients with multimorbidity at the 6-month follow-up. Integrated care may reduce care fragmentation and promote sustainability of healthcare systems. However, future efforts should be made to develop integrated care models in various settings and assess their cost-effectiveness of integrated care.

### Electronic supplementary material

Below is the link to the electronic supplementary material.

Supplementary Material 1

Supplementary Material 2

## Data Availability

Data and resources were shared with eligible researchers through established academic channels. The datasets used in this study are available from the corresponding authors upon request.

## Abbreviations

WHO: World Health Organization
IACP: Integrated ambulatory care program
TIDieR: Template for Intervention Description and Replication
NHI: National Health Insurance
CCI: Charlson Comorbidity Index
mFI: Multimorbidity Frailty Index
ED: Emergency department
GEE: Generalized Estimation Equation
NTD: New Taiwan Dollar
