# Supplementary material for: Effects of an integrated ambulatory care program on healthcare utilization and costs in older patients with multimorbidity: a propensity score-matched cohort study
Source: BMC Geriatr. 2024 Jan 29;24:109. doi: 10.1186/s12877-023-04654-y (PMC10826123; doi:10.1186/s12877-023-04654-y)
Supplement: Supplementary file 2 — Supplementary Material 2 [file 12877_2023_4654_MOESM2_ESM.docx]

Supplementary Table 2 Baseline demographic and clinical characteristics of IACP and loss to follow-up

| Variables | IACP  (n=166) | loss to follow-up  (n=16) | *p* value |
| --- | --- | --- | --- |
| **Demographic characteristics** |  |  |  |
| Age (years), mean (SD) | 77.15 (7.77) | 80.44 (6.76) | 0.104 |
| Range (min–max) | 65-94 | 70-88 |  |
| Sex, male, n (%) | 82 (49.4) | 9 (56.25) | 0.601 |
| Home to hospital distance≤ 30 minutes car drive, n (%) | 151 (91.0) | 14 (87.5) | 0.649 |
| **Clinical characteristics** |  |  |  |
| Charlson Comorbidity Index, mean (SD) | 3.72 (2.48) | 3.06 (2.11) | 0.305 |
| Chronic diseases, mean (SD) | 8.89 (3.39) | 7.88 (2.78) | 0.249 |
| BMI, mean (SD) | 24.31 (3.86) | 24.78 (5.20) | 0.648 |
| Diseases, n (%) |  |  |  |
| Diabetes | 87 (52.4) | 5 (31.3) | 0.106 |
| Diabetes with end-organ damage | 78 (47.0) | 2 (12.5) | 0.008* |
| Moderate-to-severe renal disease | 76 (45.8) | 5 (31.3) | 0.264 |
| Peptic ulcer disease | 38 (22.9) | 3 (18.8) | 1.000 |
| Cerebral vascular diseases | 36 (21.7) | 3 (18.8) | 1.000 |
| Any tumor | 25 (15.1) | 5 (31.3) | 0.096 |
| Dementia | 25 (15.1) | 2 (12.5) | 1.000 |
| Chronic pulmonary diseases | 22 (13.3) | 3 (18.8) | 0.465 |
| Congestive heart failure | 22 (13.3) | 4 (25.0) | 0.253 |
| Myocardial infarction | 4 (2.4) | 0 | 1.000 |
| Hemiplegia | 3 (1.8) | 0 | 1.000 |
| Peripheral vascular disease | 2 (1.2) | 1 (6.3) | 0.242 |
| Metastatic solid tumor | 2 (1.2) | 0 | 1.000 |
| Mild liver disease | 1 (0.6) | 1 (6.3) | 0.169 |
| Connective tissue diseases | 1 (0.6) | 0 | 1.000 |
| Moderate-to-severe liver disease | 1 (0.6) | 1 (6.3) | 0.169 |
| Lymphoma | 0 | 0 | - |
| Leukemia | 0 | 0 | - |
| AIDS | 0 | 0 | - |
